# Computed tomography-defined sarcopenia is associated with nutritional vulnerability and short-term clinical burden in hospitalized patients with lymphoma

**DOI:** 10.3389/fnut.2026.1920154

**Published:** 2026-08-17

**Authors:** Ruoxiang Sun, Hua Jiang, Xu Li, Xianping Liu, Yuxian Jian, Yanjie Fan, Dongjie Guo, Yuanqin Chen, Xiaofeng Qu, Xiuhua Sun, Xingyue Zhai

**Affiliations:** 1 Department of Clinical Nutrition, The Second Hospital of Dalian Medical University, Dalian, China; 2 School of Public Health, Dalian Medical University, Dalian, China; 3 Department of Public Health, Dalian Health Development Center, Dalian, China; 4 School of Health Preservation and Rehabilitation, Dalian Medical University, Dalian, China; 5 Department of Radiology, The Second Hospital of Dalian Medical University, Dalian, China; 6 Department of Lymphoma/Head and Neck Medical Oncology, The Second Hospital of Dalian Medical University, Dalian, China

**Keywords:** clinical outcomes, computed tomography, geriatric nutritional risk index, lymphoma, nutritional status, sarcopenia, treatment tolerance

## Abstract

**Introduction:**

Sarcopenia in lymphoma remains insufficiently characterized, particularly when defined by both computed tomography-derived skeletal muscle mass and muscle strength. This study aimed to investigate the prevalence, nutritional correlates, auxiliary discriminatory indicators, and short-term clinical implications of computed tomography-defined sarcopenia in hospitalized patients with lymphoma.

**Methods:**

This single-center cohort study included 136 hospitalized patients with lymphoma. Sarcopenia was defined as reduced skeletal muscle index at the third lumbar vertebra combined with low handgrip strength. Clinical, nutritional, laboratory, lymphoma-specific, and short-term outcome variables were compared between patients with and without sarcopenia. Multivariable logistic regression, receiver operating characteristic curve analysis, and Spearman correlation analysis were performed.

**Results:**

Computed tomography-defined sarcopenia was present in 24.26% of patients. Patients with sarcopenia more frequently had reduced recent food intake, reduced protein intake, poorer performance status, nutritional risk, and Global Leadership Initiative on Malnutrition-defined malnutrition. In the nutritional extension model, Geriatric Nutritional Risk Index below 98 (OR 8.238, 95% CI 2.748–24.690, *p* < 0.001) and reduced food intake during the previous week (OR 10.189, 95% CI 2.926–35.478, *p* < 0.001) were independently associated with sarcopenia. Geriatric Nutritional Risk Index and Patient-Generated Subjective Global Assessment showed comparable auxiliary discriminatory performance, with AUCs of 0.781 (95% CI 0.681–0.873) and 0.777 (95% CI 0.688–0.862), respectively. Baseline computed tomography-defined sarcopenia was associated with infection or febrile neutropenia (adjusted OR 2.82, 95% CI 1.20–6.60), in-hospital infection (adjusted OR 2.48, 95% CI 1.06–5.79), treatment tolerance events (adjusted OR 3.35, 95% CI 1.46–7.68), and treatment interruption (adjusted OR 2.73, 95% CI 1.09–6.82). Coexisting sarcopenia and Global Leadership Initiative on Malnutrition-defined malnutrition was observed in 16.2% of the cohort.

**Discussion:**

Computed tomography-defined sarcopenia was common in hospitalized patients with lymphoma and was associated with nutritional vulnerability, impaired treatment tolerance, and greater short-term clinical burden. These findings support integrating computed tomography-based muscle assessment with nutritional evaluation in this population.

## 1 Introduction

Globally, the incidence of lymphoma has shown a sustained upward trend (1, 2). In parallel with advances in antitumor therapy, increasing attention has been directed toward nutritional and functional impairment in patients with lymphoma. Malnutrition is common in this population and may adversely affect treatment tolerance, physical performance, and clinical outcomes. Among these complications, sarcopenia has emerged as an important concern because it reflects not only altered body composition, but also a broader decline in nutritional and functional reserve.

Sarcopenia is characterized by progressive loss of skeletal muscle mass and strength, with or without reduced physical performance (3). In patients with cancer, systemic inflammation, metabolic imbalance, reduced intake, and treatment-related burden may jointly accelerate muscle wasting (4, 5). Previous studies have shown that cancer-related sarcopenia is associated with poor quality of life, reduced treatment efficacy, and unfavorable prognosis (5, 6). Although a range of factors have been linked to sarcopenia in different malignancies, evidence specific to lymphoma remains limited (6, 7). In particular, data based on CT-defined muscle assessment and clinically accessible nutritional indicators are still insufficient.

Nutritional impairment is closely related to the development of sarcopenia. Inadequate protein and energy intake can suppress muscle protein synthesis and promote muscle breakdown, while chronic inflammation may further aggravate muscle loss (8). Several simple indicators have been used to evaluate nutritional status in clinical practice. Among them, the Geriatric Nutritional Risk Index (GNRI), derived from serum albumin and body weight, has been reported to correlate with sarcopenia and other adverse outcomes (9–11). As an easily obtainable indicator, GNRI may be useful in the early identification of patients at increased likelihood of sarcopenia. However, its value in hospitalized patients with lymphoma, especially when compared with other commonly used nutritional or screening indicators, has not been sufficiently clarified.

Although sarcopenia has been increasingly studied in patients with solid tumors, evidence specific to lymphoma remains limited. Existing studies in hematologic malignancies have largely focused on muscle mass alone or long-term survival, whereas less is known about whether a clinically defined sarcopenia phenotype, integrating CT-derived muscle mass and muscle strength, is associated with nutritional vulnerability, treatment delivery, and short-term inpatient outcomes in hospitalized patients with lymphoma. This evidence gap is clinically important because patients with lymphoma often undergo repeated hospitalizations and intensive systemic therapy, during which impaired nutritional and functional reserve may influence infection risk, treatment tolerance, and hospitalization burden.

Therefore, the present study aimed to investigate the prevalence of CT-defined sarcopenia in hospitalized patients with lymphoma, identify nutritional and lymphoma-specific factors associated with this phenotype, and evaluate the auxiliary discriminatory performance of clinically accessible nutritional and screening indicators. We further examined whether CT-defined sarcopenia, alone and in combination with GLIM-defined malnutrition, was associated with short-term clinical outcomes, including infectious complications, treatment tolerance events, treatment interruption, and cumulative hospitalization burden.

## 2 Materials and methods

### 2.1 Participants

This single-center cohort study consecutively enrolled eligible adult patients who were hospitalized in the Lymphoma Ward of the Second Hospital of Dalian Medical University between September 23 and December 1, 2025. All participants were aged 18 years or older and underwent nutritional screening, assessment of sarcopenia, and laboratory testing during hospitalization. Baseline sarcopenia, nutritional status, clinical characteristics, and laboratory parameters were assessed during hospitalization. Short-term clinical outcomes were subsequently ascertained from inpatient records and follow-up information within the predefined observation period. Patients were excluded if they had severe neurological or muscular disorders that could interfere with physical assessment, marked short-term changes in body composition within the preceding 3 months, severe cardiac, pulmonary, renal, or hepatic dysfunction, secondary malignancies, psychiatric or other conditions that precluded completion of the examinations or questionnaires, or refusal to provide informed consent. The study protocol was approved by the Ethics Committee of the Second Hospital of Dalian Medical University (Approval No. KY2025-410-02), and written informed consent was obtained from all participants before study-related assessment.

### 2.2 Sample size estimation

The sample size was initially estimated for the baseline assessment of sarcopenia and malnutrition prevalence. On the basis of previously reported prevalences of sarcopenia and malnutrition in patients with lymphoma, the preliminary minimum sample sizes required at a 95% confidence level were 121 and 111, respectively. The larger estimate was adopted. After allowance for a potential 5% attrition rate, the minimum required sample size was approximately 127. Considering the feasibility of recruitment during the study period and the planned multivariable analysis, the target sample size was set at 136, and 136 patients were ultimately included in the final analysis.

### 2.3 Assessment of sarcopenia

Sarcopenia was assessed using computed tomography (CT) imaging. SliceOmatic software (version 5.0) was used for image analysis. Skeletal muscle was segmented on axial CT images at the level of the third lumbar vertebra (L3) using tissue-specific Hounsfield unit thresholds ranging from −29 to +150. The skeletal muscle area (SMA, cm^2^) was measured, and the skeletal muscle index (SMI, cm^2^/m^2^) was calculated by normalizing SMA to the square of body height.

Although magnetic resonance imaging and CT are regarded as reference methods for the assessment of muscle mass, current guidelines, including those of the Asian Working Group for Sarcopenia, do not provide specific diagnostic cut-off values for these imaging modalities (12). Bioelectrical impedance analysis is widely used in clinical practice, but its accuracy in patients with cancer may be affected by hydration status, age, and especially edema (13, 14). By contrast, CT enables a more direct and stable assessment of skeletal muscle area and density and is less susceptible to the influence of fluid imbalance.

**Figure 1:**
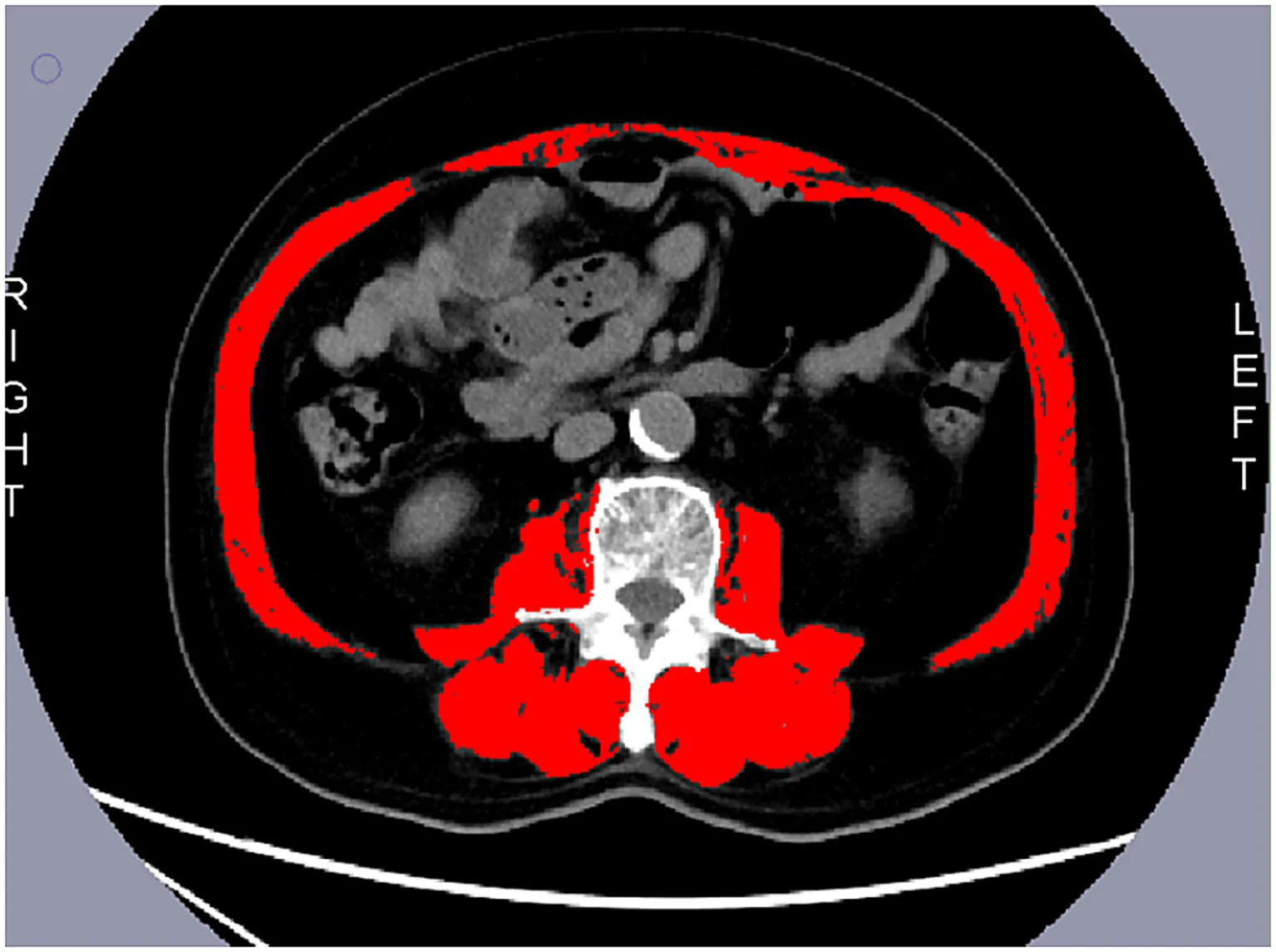
Representative axial computed tomography image at the level of the third lumbar vertebra for skeletal muscle analysis. The skeletal muscle area was 104.2 cm^2^, and the skeletal muscle index was 40.7 cm^2^/m^2^.

Accordingly, sarcopenia in the present study was defined on the basis of reduced skeletal muscle mass combined with low handgrip strength. According to the cut-off values adopted in the original study protocol (15), reduced skeletal muscle mass was defined as an SMI below 40.8 cm^2^/m^2^ for men and below 34.9 cm^2^/m^2^ for women, while low muscle strength was defined as handgrip strength below 28 kg for men and below 18 kg for women. Patients who met both criteria were diagnosed with sarcopenia (Figure 1). These CT-SMI cut-off values were selected because lymphoma-specific validated CT-based thresholds for Chinese patients were not available at the time of protocol development. The thresholds were derived from a large Chinese oncologic cohort (15) and were considered the most applicable available reference for an Asian cancer population. Therefore, they were adopted as a pragmatic reference for defining low skeletal muscle mass in the present study, together with the Asian Working Group for Sarcopenia 2019 handgrip strength criteria (12).

### 2.4 Measurement of covariates

Data on potential covariates were collected from medical records, physical examination, and nutritional assessment. The variables included sex, age, body mass index (BMI), tumor stage, payment method, involuntary weight loss, reduced food intake during the previous week, reduced protein intake, and common chronic comorbidities. In addition to general demographic, clinical, and nutritional covariates, lymphoma-specific variables were extracted from medical records, including lymphoma subtype, Ann Arbor stage, B symptoms, extranodal involvement, bone marrow involvement, serum lactate dehydrogenase status, and treatment status. Advanced disease stage was defined as Ann Arbor stage III or IV. Elevated LDH was defined according to the upper limit of the local laboratory reference range.

Handgrip strength and calf circumference were recorded as indicators of muscle function and nutritional status. Sarcopenia screening was performed with the SARC-F and SARC-CalF questionnaires, with a SARC-F score of 4 or higher and a SARC-CalF score of 11 or higher indicating a positive screening result. Comorbidity burden was assessed with the Charlson Comorbidity Index (CCI). Functional status and activities of daily living were evaluated with the Eastern Cooperative Oncology Group (ECOG) performance status scale, in which a higher score indicates poorer functional capacity.

Nutritional status was assessed with NRS-2002, PG-SGA, GLIM-defined malnutrition, and the Geriatric Nutritional Risk Index (GNRI). GNRI was calculated as follows: 1.489 × serum albumin (g/L) + 41.7 × (actual body weight/ideal body weight). In addition, inflammatory and nutrition-related indices were derived from routine laboratory data, including the systemic immune-inflammation index (SII), calculated as neutrophil count × platelet count/lymphocyte count, the platelet-to-lymphocyte ratio (PLR), calculated as platelet count/lymphocyte count, the neutrophil-to-lymphocyte ratio (NLR), calculated as neutrophil count/lymphocyte count, and the prognostic nutritional index (PNI), calculated as serum albumin (g/L) + 5 × lymphocyte count (10^9^/L).

In the secondary analyses, GNRI, PNI, PG-SGA, SARC-F, and calf circumference were further evaluated as simple clinical indicators for the identification of CT-defined sarcopenia. Coexisting sarcopenia and malnutrition was defined as the simultaneous presence of CT-defined sarcopenia and GLIM-defined malnutrition. However, detailed treatment phase and line of therapy, such as newly diagnosed disease, relapse, first-line therapy, or later-line therapy, were not prospectively collected in a standardized manner in the original protocol and could not be reliably categorized for all participants. Therefore, these variables were not included in the primary adjusted analyses.

### 2.5 Laboratory tests

Fasting venous blood samples were collected in the morning within 24 h after hospital admission after an overnight fast of at least 12 h. Laboratory parameters obtained from routine blood testing included C-reactive protein (CRP), white blood cell count (WBC), neutrophil count (NEU), lymphocyte count (LYM), platelet count (PLT), serum albumin (ALB), and serum creatinine (SCR).

### 2.6 Assessment of short-term clinical outcomes

Short-term clinical outcomes were ascertained from inpatient records and follow-up information after baseline assessment and within the predefined observation period. These outcomes included cumulative inpatient days during the observation period, prolonged cumulative hospitalization exceeding 60 days, in-hospital infection, febrile neutropenia, the composite outcome of infection or febrile neutropenia, severe complications, antibiotic escalation, chemotherapy delay, chemotherapy dose reduction, treatment interruption, treatment tolerance events, transfer to the intensive care unit, and 30-day and 90-day mortality. Cumulative inpatient days were calculated as the total number of inpatient days during the observation period, including repeated admissions when applicable. Prolonged cumulative hospitalization was defined as cumulative inpatient days exceeding 60 days. Febrile neutropenia and in-hospital infection were identified according to clinical diagnoses documented in the medical records. The composite outcome of infection or febrile neutropenia was used to capture clinically relevant infectious complications during hospitalization. Treatment tolerance events were defined as the occurrence of chemotherapy delay, chemotherapy dose reduction, or treatment interruption during the observation period. These outcomes were selected to reflect short-term clinical burden, treatment delivery, and acute deterioration in hospitalized patients with lymphoma.

### 2.7 Statistical analysis

Statistical analyses were performed with SPSS (version 26.0) and R software (version 4.0.3). Continuous variables were expressed as mean ± standard deviation or median (interquartile range), as appropriate, and categorical variables were expressed as number (percentage). For comparisons between patients with and without CT-defined sarcopenia, the independent-samples *t* test was used for approximately normally distributed continuous variables, the Mann–Whitney U test was used for non-normally distributed continuous variables, and the chi-square test or Fisher exact test was used for categorical variables. The ROC analysis was exploratory and focused on the magnitude and precision of the AUC estimates rather than formal pairwise testing between curves.

To examine factors associated with CT-defined sarcopenia, two multivariable logistic regression models were constructed. The base clinical model included body mass index, reduced food intake during the previous week, ECOG performance status of 2 or higher, type 2 diabetes mellitus, and hypertension. The nutritional extension model replaced body mass index with GNRI below 98 while retaining the other clinically relevant variables. Odds ratios and 95% confidence intervals were reported. Model fit was evaluated using the Akaike information criterion, the Bayesian information criterion, and pseudo *R*^2^.

Receiver operating characteristic curve analysis was used to evaluate the auxiliary discriminatory performance of GNRI, PNI, PG-SGA, SARC-F, and calf circumference for CT-defined sarcopenia. The area under the curve, 95% confidence interval, optimal cut-off value, sensitivity, specificity, and Youden index were calculated for each indicator. Spearman correlation analysis was performed to examine the correlations of GNRI and PNI with skeletal muscle index and handgrip strength. The prevalence of coexisting CT-defined sarcopenia and GLIM-defined malnutrition was also described.

For short-term clinical outcomes, between-group comparisons were first performed according to CT-defined sarcopenia status. Multivariable logistic regression models were then used to examine the associations between CT-defined sarcopenia and selected binary outcomes, including infection or febrile neutropenia, in-hospital infection, treatment tolerance events, treatment interruption, and prolonged cumulative hospitalization exceeding 60 days. The primary adjusted outcome model included age, sex, advanced Ann Arbor stage, and elevated LDH. Additional sensitivity models further adjusted for GNRI or GLIM-defined malnutrition to assess whether the associations were attenuated after accounting for nutritional status. For cumulative inpatient days, log-transformed values were analyzed in linear regression models when appropriate. Results were reported as odds ratios or regression coefficients with 95% confidence intervals.

In exploratory analyses, patients were further classified into four groups according to the presence or absence of CT-defined sarcopenia and GLIM-defined malnutrition. Event rates and cumulative inpatient days were compared across these groups to describe the joint clinical burden associated with sarcopenia and malnutrition. Given the design, all findings were interpreted as associations rather than causal effects. Analyses were performed on available cases for each variable. All tests were two-sided, and a *p* value of less than 0.05 was considered statistically significant.

## 3 Results

### 3.1 Study population characteristics

The final study cohort comprised 136 hospitalized patients with lymphoma, including 77 men and 59 women, with a mean age of 62.68 years. Among all participants, the prevalence of CT-defined sarcopenia was 24.26% (33/136). In the overall cohort, 32.35% of patients were classified as being at nutritional risk according to NRS-2002, and 32.35% met the criteria for GLIM-defined malnutrition.

Compared with patients without sarcopenia, those with sarcopenia were more likely to have lower body mass index, involuntary weight loss, reduced food intake during the previous week, reduced protein intake, poorer functional status, and a higher prevalence of metabolic comorbidities, particularly type 2 diabetes mellitus and hypertension. By contrast, no significant between-group differences were observed for sex, age category, payment method, tumor stage, coronary heart disease, hypothyroidism, or Charlson Comorbidity Index category. Detailed baseline characteristics are presented in Table 1.

**Table 1:** Baseline characteristics of the study participants.

Characteristic	Group		Statistical value	*p*
	SAR(+) (*n* = 33)	SAR(−) (*n* = 103)		
Sex, *n* (%)			χ^2^ = 0.87	0.35
Male	21 (63.6)	56 (54.4)		
Female	12 (36.4)	47 (45.6)		
Age (years)	65.88 ± 14.27	61.65 ± 12.43	1.64	0.10
≥60	26 (78.8)	66 (64.1)	χ^2^ = 2.471	0.12
<60	7 (21.2)	37 (35.9)		
Payment method			χ^2^ = 2.92	0.23
Urban employee basic medical insurance	5 (15.2)	27 (26.2)		
Urban and rural resident basic medical insurance	28 (84.8)	73 (70.9)		
Other	0 (0)	3 (2.9)		
Clinical characteristics				
Involuntary weight loss	29 (87.9)	34 (33)	χ^2^ = 30.26	<0.001
Reduced food intake in the past week	28 (84.8)	29 (28.2)	χ^2^ = 33.00	<0.001
Reduced protein intake	28 (84.8)	35 (34)	χ^2^ = 26.01	<0.001
ECOG score ≥2	16 (48.5)	13 (12.6)	χ^2^ = 19.16	<0.001
Tumor stage			χ^2^ = 0.33	0.57
Terminal stage	15 (45.5)	41 (39.8)		
Non-terminal stage	18 (54.5)	62 (60.2)		
Comorbidities				
T2DM	12 (26.8)	11 (10.7)	χ^2^ = 11.73	<0.001
Hypertension	11 (33.3)	15 (14.6)	χ^2^ = 5.70	0.02
Coronary heart disease (CHD)	2 (6.1)	4 (3.9)	/	0.63
Hypothyroidism	1 (3)	4 (3.9)	/	1.00
Charlson comorbidity index (CCI) score			χ^2^ = 0.39	0.92
1–3	24 (72.7)	78 (75.7)		
4–5	8 (24.2)	22 (21.4)		
>5	1 (3.0)	3 (2.9)		
NRS-2002 score ≥3	21 (63.6)	23 (22.3)	17.64	<0.001
GLIM-Defined Malnutrition (+)	22 (66.7)	22 (21.4)	21.42	<0.001

Data are presented as mean ± standard deviation, median (interquartile range), or number (percentage).

### 3.2 Factors associated with CT-defined sarcopenia

To further examine factors associated with CT-defined sarcopenia, two multivariable logistic regression models were constructed. In the base clinical model, lower body mass index and reduced food intake during the previous week were significantly associated with CT-defined sarcopenia. Specifically, each one-unit increase in body mass index was associated with lower odds of sarcopenia, whereas reduced food intake during the previous week was associated with a markedly higher likelihood of sarcopenia. ECOG performance status of 2 or higher, type 2 diabetes mellitus, and hypertension were retained in the model as clinically relevant variables, although they did not reach statistical significance.

In the nutritional extension model, body mass index was replaced by GNRI <98. In this model, GNRI below 98 and reduced food intake during the previous week remained significantly associated with CT-defined sarcopenia. By contrast, ECOG performance status of 2 or higher, type 2 diabetes mellitus, and hypertension were not significantly associated with sarcopenia after adjustment. Compared with the base clinical model, the nutritional extension model showed better overall fit, with lower AIC and BIC values and a higher pseudo *R*^2^. The detailed results of both models are presented in Tables 2, 3.

**Table 2:** Multivariable logistic regression models for factors associated with CT-defined sarcopenia.

Variable	*β*	SE	Wald *χ^2^*	*p-*value	OR	95% CI
Model 1. Base clinical model						
BMI	−0.154	0.073	4.416	0.036	0.857	0.743–0.990
Reduced Food Intake in the Past Week	2.661	0.615	18.742	<0.001	14.306	4.289–47.716
ECOG Score ≥2	0.810	0.549	2.173	0.140	2.248	0.766–6.597
T2DM	1.105	0.708	2.439	0.118	3.019	0.754–12.086
Hypertension	−0.977	0.744	1.725	0.189	0.376	0.088–1.618
Intercept	−0.314	1.676	0.035	0.851	0.730	
Model 2. Nutritional extension						
GNRI <98	2.109	0.560	14.176	<0.001	8.238	2.748–24.690
Reduced food intake in the past week	2.321	0.637	13.299	<0.001	10.189	2.926–35.478
ECOG score ≥2	0.282	0.595	0.225	0.635	1.326	0.413–4.253
T2DM	0.959	0.753	1.620	0.203	2.609	0.596–11.422
Hypertension	−1.519	0.816	3.465	0.063	0.219	0.044–1.084
Intercept	−1.772	0.707	6.284	0.012	0.170	

Model 1 fit: *n* = 136, AIC = 112.503, BIC = 129.979, Pseudo *R*^2^ = 0.333; Model 2 fit: *n* = 136, AIC = 101.791, BIC = 119.267, Pseudo *R*^2^ = 0.404.

BMI, body mass index; ECOG, Eastern Cooperative Oncology Group; GNRI, Geriatric Nutritional Risk Index; T2DM, type 2 diabetes mellitus; OR, odds ratio; CI, confidence interval.

**Table 3:** Discriminatory performance of nutritional and screening indicators for CT-defined sarcopenia.

Indicator	Optimal cut-off value	Sensitivity (%)	Specificity (%)	Youden’s index	AUC (95% CI)
GNRI	97.88	75.8	81.4	0.571	0.781 (0.681–0.873)
PG-SGA	8	75.8	68.9	0.447	0.777 (0.688–0.862)
SARC-F	3	63.6	82.5	0.462	0.716 (0.601–0.819)
Calf circumference	32.0 cm	66.7	68.0	0.346	0.700 (0.604–0.795)
PNI	47.15	66.7	59.2	0.259	0.629 (0.520–0.734)

Given the design, these findings should be interpreted as associations rather than causal relationships. Overall, reduced food intake and lower GNRI may help identify hospitalized patients with lymphoma who are more likely to have CT-defined sarcopenia.

### 3.3 Discriminatory performance of nutritional and screening indicators for CT-defined sarcopenia

Receiver operating characteristic curve analysis was performed to compare the auxiliary discriminatory performance of several clinically accessible nutritional and screening indicators for CT-defined sarcopenia. GNRI had the numerically highest AUC, with an area under the curve of 0.781 (95% CI 0.681–0.873), an optimal cut-off value of 97.88, a sensitivity of 75.8%, a specificity of 81.4%, and a Youden index of 0.571. PG-SGA showed a similar level of discriminatory performance, with an area under the curve of 0.777 (95% CI 0.688–0.862), an optimal cut-off value of 8, a sensitivity of 75.8%, a specificity of 68.9%, and a Youden index of 0.447.

Among the remaining indicators, SARC-F showed moderate discriminatory ability, with an area under the curve of 0.716 (95% CI 0.601–0.819), a sensitivity of 63.6%, a specificity of 82.5%, and a Youden index of 0.462 at the optimal cut-off value of 3. Calf circumference also showed moderate discriminatory ability, with an area under the curve of 0.700 (95% CI 0.604–0.795), a sensitivity of 66.7%, a specificity of 68.0%, and a Youden index of 0.346 at the optimal cut-off value of 32.0 cm. By contrast, the discriminatory performance of PNI was relatively weak, with an area under the curve of 0.629 (95% CI 0.520–0.734), a sensitivity of 66.7%, a specificity of 59.2%, and a Youden index of 0.259 at the optimal cut-off value of 47.15.

**Figure 2:**
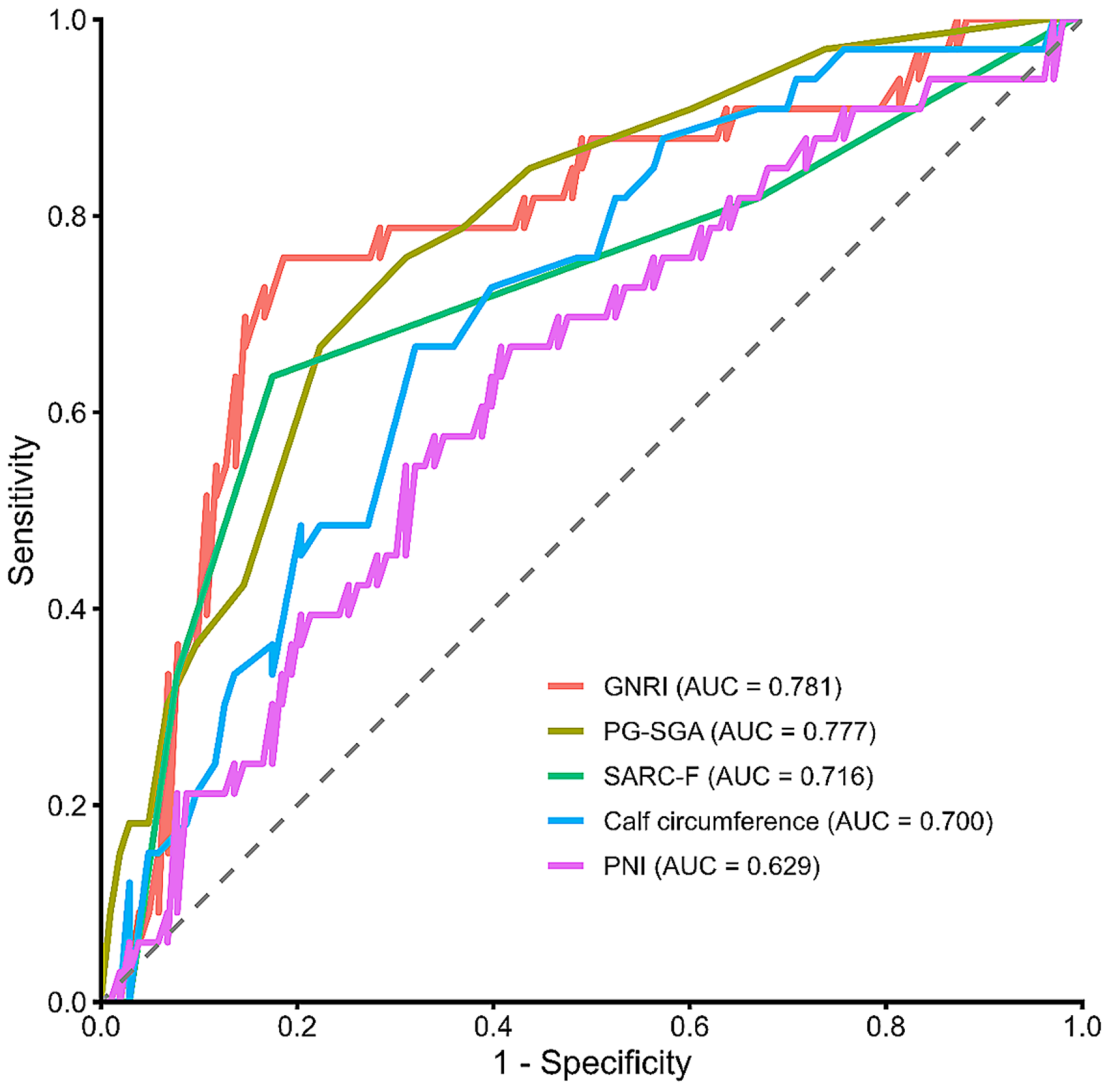
Receiver operating characteristic curves of clinically accessible nutritional and screening indicators for identifying computed tomography-defined sarcopenia. The evaluated indicators included the Geriatric Nutritional Risk Index, Patient-Generated Subjective Global Assessment, SARC-F, calf circumference, and Prognostic Nutritional Index. AUC, area under the curve; GNRI, Geriatric Nutritional Risk Index; PG-SGA, Patient-Generated Subjective Global Assessment; PNI, Prognostic Nutritional Index.

Taken together, these findings indicate that GNRI and PG-SGA showed comparable and relatively favorable auxiliary discriminatory performance among the evaluated indicators for identifying patients with CT-defined sarcopenia, whereas SARC-F and calf circumference may still provide useful supportive information in routine clinical assessment. The corresponding receiver operating characteristic curves are shown in Figure 2.

### 3.4 Correlations of GNRI and PNI with SMI and handgrip strength

Spearman correlation analysis was performed to further examine the relationships of GNRI and PNI with skeletal muscle index and handgrip strength. GNRI was positively correlated with skeletal muscle index (*r* = 0.202, *p* = 0.019) and with handgrip strength (*r* = 0.296, *p* < 0.001), indicating that better nutritional status, as reflected by a higher GNRI, was associated with greater muscle mass and better muscle strength.

**Figure 3:**
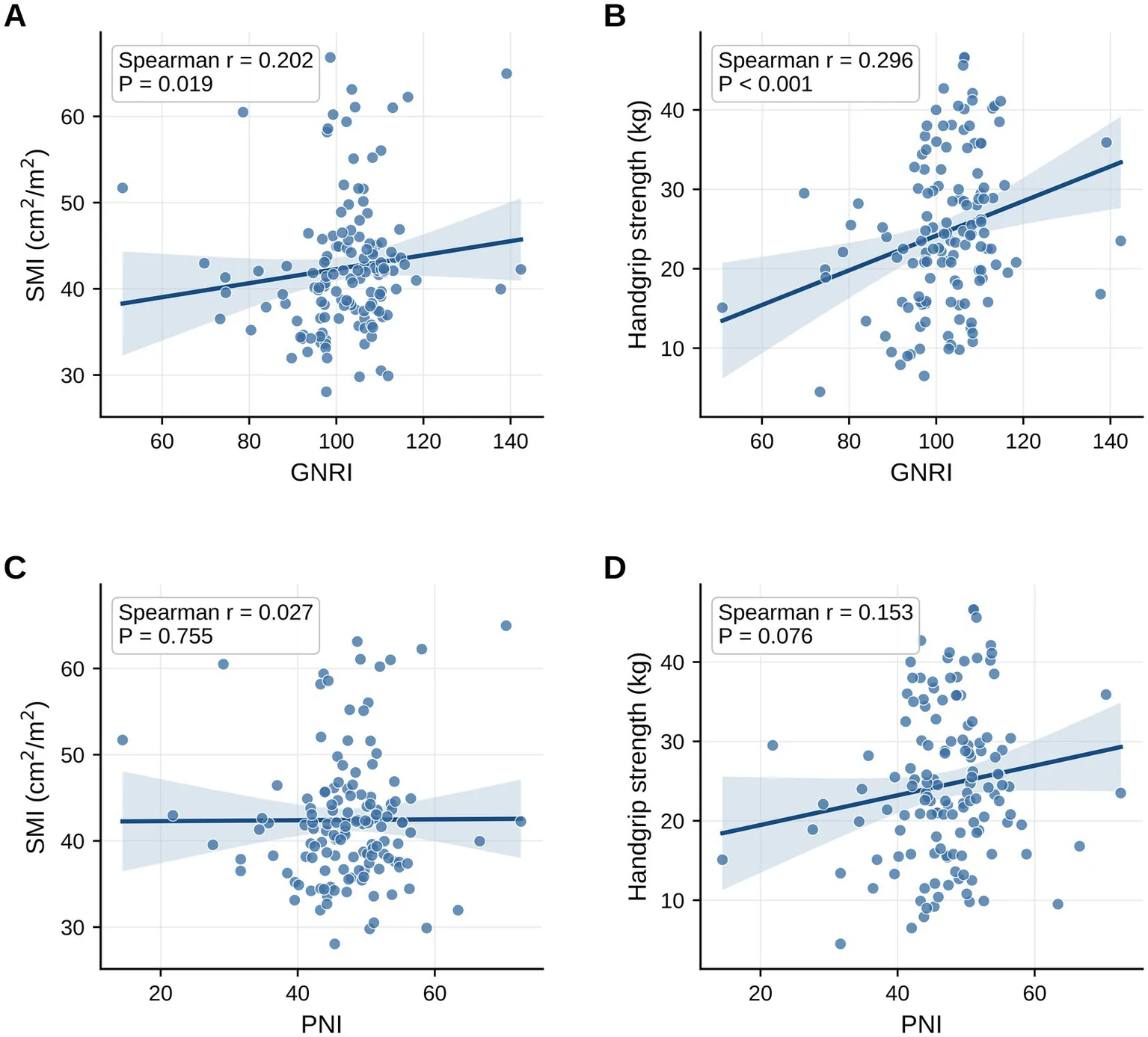
Scatter plots showing the relationships of the Geriatric Nutritional Risk Index and Prognostic Nutritional Index with skeletal muscle index and handgrip strength. **(A)** Geriatric Nutritional Risk Index versus skeletal muscle index; **(B)** Geriatric Nutritional Risk Index versus handgrip strength; **(C)** Prognostic Nutritional Index versus skeletal muscle index; and **(D)** Prognostic Nutritional Index versus handgrip strength. Each point represents one participant. The solid line indicates the fitted trend, and the shaded area represents the 95% confidence interval. GNRI, Geriatric Nutritional Risk Index; PNI, Prognostic Nutritional Index; SMI, skeletal muscle index.

By contrast, PNI was not significantly correlated with skeletal muscle index (*r* = 0.027, *p* = 0.755) or handgrip strength (*r* = 0.153, *p* = 0.076). These findings further support the value of GNRI as a more informative nutritional indicator than PNI for the assessment of CT-defined sarcopenia in this cohort. The detailed correlation results are presented in Table 4. These relationships are visually illustrated in the scatter plots, which show positive trends for GNRI with both skeletal muscle index and handgrip strength, whereas the corresponding associations for PNI appear weak and non-significant (Figure 3).

**Table 4:** Correlations of GNRI and PNI with SMI and handgrip strength.

Indicator	Outcome	Spearman *r*	*p*
GNRI	SMI	0.202	0.019
GNRI	Handgrip strength	0.296	<0.001
PNI	SMI	0.027	0.755
PNI	Handgrip strength	0.153	0.076

### 3.5 Coexistence of CT-defined sarcopenia and GLIM-defined malnutrition

Nutritional impairment was more frequent among patients with CT-defined sarcopenia than among those without sarcopenia. In the sarcopenia group, 21 patients (63.6%) had an NRS-2002 score of 3 or higher, compared with 23 patients (22.3%) in the non-sarcopenia group. Similarly, GLIM-defined malnutrition was present in 22 patients (66.7%) with sarcopenia and in 22 patients (21.4%) without sarcopenia.

Overall, 22 patients had coexisting CT-defined sarcopenia and GLIM-defined malnutrition, accounting for 16.2% of the total cohort. This overlap suggested that sarcopenia and malnutrition were not isolated conditions in hospitalized patients with lymphoma, and provided a basis for further evaluating the joint clinical burden of these two conditions.

### 3.6 Short-term clinical outcomes according to CT-defined sarcopenia

Patients with CT-defined sarcopenia had a higher short-term clinical burden than those without sarcopenia. The rate of infection or febrile neutropenia was 63.6% in the sarcopenia group and 37.9% in the non-sarcopenia group (*p* = 0.009). Similar between-group differences were observed for in-hospital infection (51.5% vs. 28.2%, *p* = 0.014), treatment tolerance events (51.5% vs. 24.3%, *p* = 0.003), and treatment interruption (33.3% vs. 16.5%, *p* = 0.037).

Cumulative inpatient days were also longer in patients with sarcopenia than in those without sarcopenia, with median values of 56.0 days (45.0–99.0) and 39.0 days (25.0–71.0), respectively (*p* = 0.017). Prolonged cumulative hospitalization exceeding 60 days was more frequent in the sarcopenia group (45.5% vs. 31.1%), although this difference did not reach statistical significance (*p* = 0.130). These findings indicate that CT-defined sarcopenia was accompanied by a greater burden of infectious events, impaired treatment delivery, and longer hospitalization in this cohort. The detailed results are presented in Table 5.

**Table 5:** Short-term clinical outcomes according to CT-defined sarcopenia status.

Outcome	Overall	Non-sarcopenia	Sarcopenia	P value
Cumulative inpatient days	47.00 (26.00, 85.50)	39.00 (25.00, 71.00)	56.00 (45.00, 99.00)	0.017
Prolonged hospitalization >60 days	47 (34.6%)	32 (31.1%)	15 (45.5%)	0.130
In-hospital infection	46 (33.8%)	29 (28.2%)	17 (51.5%)	0.014
Febrile neutropenia	40 (29.4%)	28 (27.2%)	12 (36.4%)	0.314
Infection or febrile neutropenia	60 (44.1%)	39 (37.9%)	21 (63.6%)	0.009
Severe complication	37 (27.2%)	27 (26.2%)	10 (30.3%)	0.646
Antibiotic escalation	38 (27.9%)	26 (25.2%)	12 (36.4%)	0.215
Chemotherapy delay	20 (14.7%)	13 (12.6%)	7 (21.2%)	0.261
Chemotherapy dose reduction	11 (8.1%)	7 (6.8%)	4 (12.1%)	0.461
Treatment interruption	28 (20.6%)	17 (16.5%)	11 (33.3%)	0.037
Treatment tolerance event	42 (30.9%)	25 (24.3%)	17 (51.5%)	0.003
Composite adverse inpatient outcome	88 (64.7%)	64 (62.1%)	24 (72.7%)	0.268
ICU transfer	7 (5.1%)	5 (4.9%)	2 (6.1%)	0.677
30-day mortality	7 (5.1%)	5 (4.9%)	2 (6.1%)	0.677
90-day mortality	11 (8.1%)	10 (9.7%)	1 (3.0%)	0.295

### 3.7 CT-defined sarcopenia and short-term adverse clinical outcomes

Multivariable logistic regression was further performed to evaluate the association between CT-defined sarcopenia and selected adverse clinical outcomes. In the primary adjusted model, which included age, sex, advanced Ann Arbor stage, and elevated LDH, CT-defined sarcopenia was associated with higher odds of infection or febrile neutropenia (adjusted OR 2.82, 95% CI 1.20–6.60, *p* = 0.017), in-hospital infection (adjusted OR 2.48, 95% CI 1.06–5.79, *p* = 0.036), treatment tolerance events (adjusted OR 3.35, 95% CI 1.46–7.68, *p* = 0.004), and treatment interruption (adjusted OR 2.73, 95% CI 1.09–6.82, *p* = 0.031). The association with prolonged cumulative hospitalization exceeding 60 days was in the same direction but did not reach statistical significance (adjusted OR 2.08, 95% CI 0.91–4.78, *p* = 0.085).

**Figure 4:**
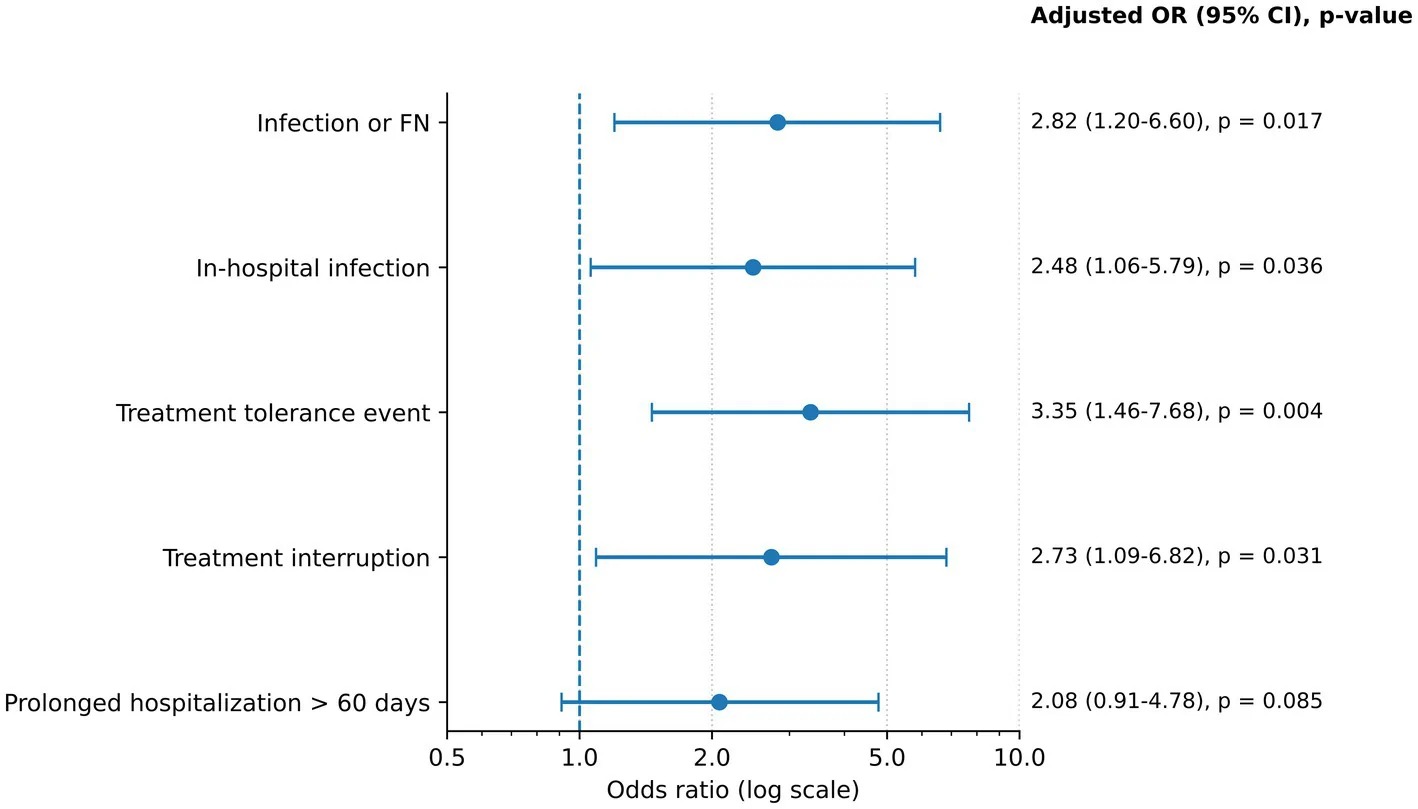
Association between computed tomography-defined sarcopenia and adverse clinical outcomes. Odds ratios were derived from multivariable logistic regression models adjusted for age, sex, advanced Ann Arbor stage, and elevated lactate dehydrogenase. FN, febrile neutropenia; OR, odds ratio; CI, confidence interval; LDH, lactate dehydrogenase. Model adjusted for age, sex, advanced Ann Arbor stage, and elevated LDH.

Sensitivity analyses showed that the association between CT-defined sarcopenia and treatment tolerance events remained statistically significant after additional adjustment for GNRI (adjusted OR 3.38, 95% CI 1.41–8.13, *p* = 0.006) and after additional adjustment for GLIM-defined malnutrition (adjusted OR 2.54, 95% CI 1.02–6.34, *p* = 0.046). By contrast, the associations with infection-related outcomes were attenuated after additional adjustment for nutritional status. These results suggest that impaired nutritional reserve may partly account for the relationship between sarcopenia and infectious complications, whereas the association with treatment tolerance events appeared more stable. The main adjusted model is summarized in Table 6 and illustrated in Figure 4. The complete sensitivity analyses are provided in Supplementary Table S1.

**Table 6:** Association between CT-defined sarcopenia and adverse clinical outcomes.

Outcome	Model	Adjusted OR	95% CI	*p*-value	*N*	Events	AIC	BIC	Pseudo R^2^
Infection or febrile neutropenia	Model 2	2.82	1.20–6.60	*p* = 0.017	136	60	180.23	197.71	0.099
In-hospital infection	Model 2	2.48	1.06–5.79	*p* = 0.036	136	46	169.16	186.63	0.097
Treatment tolerance event	Model 2	3.35	1.46–7.68	*p* = 0.004	136	42	171.44	188.91	0.052
Treatment interruption	Model 2	2.73	1.09–6.82	*p* = 0.031	136	28	143.99	161.46	0.046
Prolonged hospitalization >60 days	Model 2	2.08	0.91–4.78	*p* = 0.085	136	47	180.5	197.97	0.039

Model adjusted for age, sex, advanced Ann Arbor stage, and elevated LDH. FN, febrile neutropenia; OR, odds ratio; CI, confidence interval; LDH, lactate dehydrogenase.

### 3.8 Joint clinical burden of CT-defined sarcopenia and GLIM-defined malnutrition

To further describe the combined clinical burden of sarcopenia and malnutrition, patients were classified into four groups according to CT-defined sarcopenia and GLIM-defined malnutrition status. The four groups included 81 patients (59.6%) without sarcopenia or malnutrition, 22 patients (16.2%) without sarcopenia but with malnutrition, 11 patients (8.1%) with sarcopenia but without malnutrition, and 22 patients (16.2%) with both conditions.

**Figure 5:**
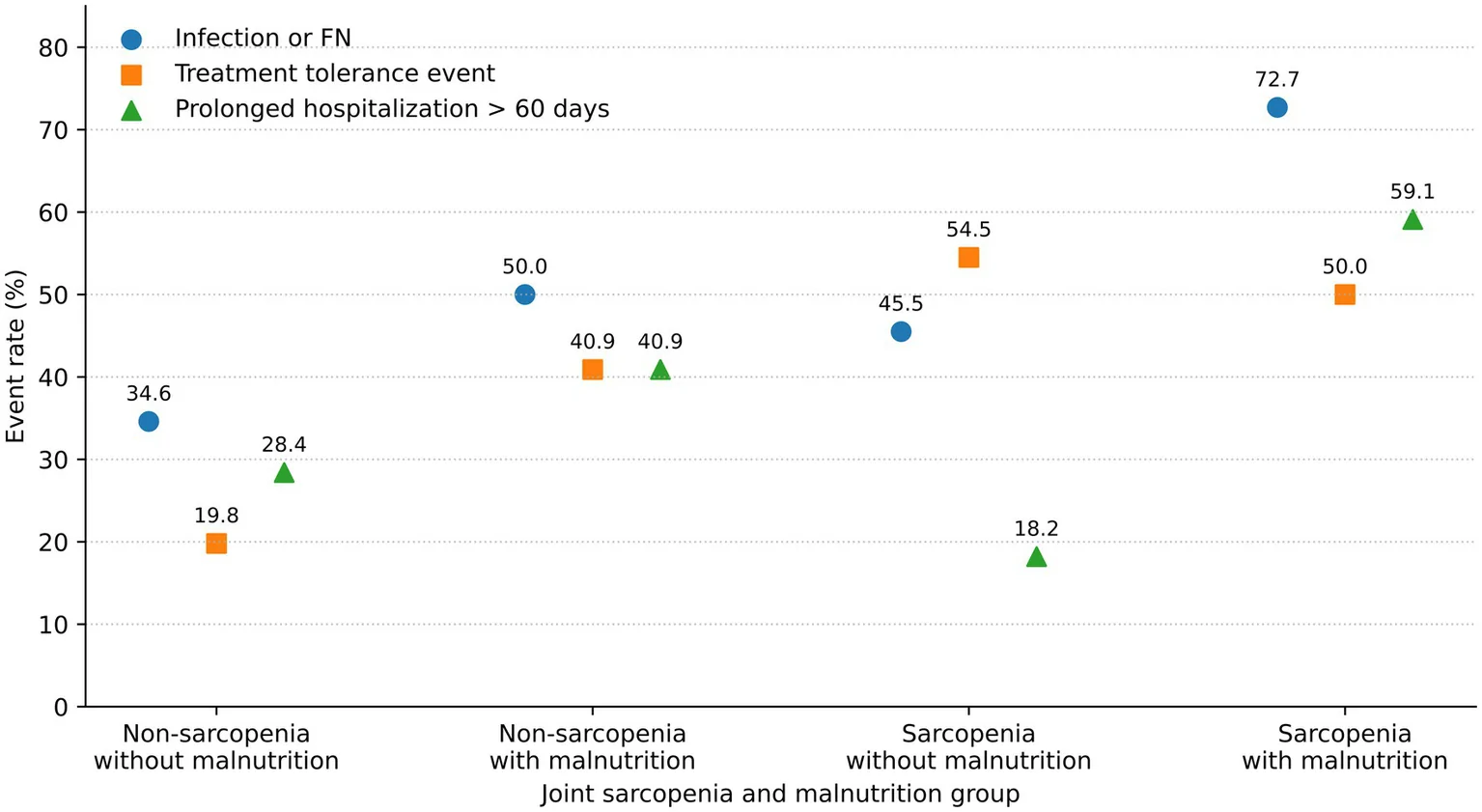
Clinical outcomes across joint groups defined by computed tomography-defined sarcopenia and Global Leadership Initiative on Malnutrition-defined malnutrition. Event rates are shown for infection or febrile neutropenia, treatment tolerance events, and prolonged cumulative hospitalization exceeding 60 days. FN, febrile neutropenia; GLIM, Global Leadership Initiative on Malnutrition, Event rates are shown across four groups defined by CT-defined sarcopenia and GLIM-defined malnutrition.

Patients with coexisting CT-defined sarcopenia and GLIM-defined malnutrition had the highest rate of infection or febrile neutropenia (72.7%) and prolonged cumulative hospitalization exceeding 60 days (59.1%). This group also had the longest cumulative inpatient stay, with a median of 72.5 days (48.50–104.75). Treatment tolerance events were frequent in both sarcopenia groups, occurring in 54.5% of patients with sarcopenia alone and in 50.0% of patients with coexisting sarcopenia and malnutrition. These findings suggest that the coexistence of sarcopenia and malnutrition marked a subgroup with a particularly high infectious and hospitalization burden, while treatment tolerance events appeared to be closely related to sarcopenia status itself. The joint group results are shown in Table 7 and Figure 5.

**Table 7:** Clinical outcomes across joint sarcopenia and GLIM-defined malnutrition groups.

Group	*N*	Percentage	Infection or FN, %	In-hospital infection, %	Treatment tolerance event, %	Treatment interruption, %	Prolonged hospitalization >60 days, %	Composite adverse inpatient outcome, %	Cumulative inpatient days, median (IQR)
Non-sarcopenia without malnutrition	81	59.6	34.6	24.7	19.8	12.3	28.4	59.3	38.0 (25.00–69.00)
Non-sarcopenia with malnutrition	22	16.2	50	40.9	40.9	31.8	40.9	72.7	47.0 (26.00–81.00)
Sarcopenia without malnutrition	11	8.1	45.5	45.5	54.5	27.3	18.2	54.5	47.0 (36.50–55.00)
Sarcopenia with malnutrition	22	16.2	72.7	54.5	50	36.4	59.1	81.8	72.5 (48.50–104.75)

## 4 Discussion

In this single-center hospital-based cohort study of hospitalized patients with lymphoma, CT-defined sarcopenia was identified in nearly one quarter of the cohort and was closely linked to nutritional vulnerability, impaired functional reserve, and a higher short-term clinical burden. Reduced recent food intake and lower nutritional reserve, particularly GNRI below 98, were independently associated with CT-defined sarcopenia. Among the evaluated nutritional and screening indicators, GNRI and PG-SGA showed comparable and relatively favorable auxiliary discriminatory performance. Beyond nutritional assessment, CT-defined sarcopenia was associated with higher rates of infection or febrile neutropenia, in-hospital infection, treatment tolerance events, treatment interruption, and longer cumulative hospitalization. In the adjusted analyses, the association with treatment tolerance events remained relatively stable after further accounting for nutritional status. These findings suggest that CT-defined sarcopenia may represent more than a body composition phenotype in hospitalized patients with lymphoma; it may also identify a clinically vulnerable subgroup with nutritional compromise and poorer short-term treatment-related outcomes.

### 4.1 CT-defined sarcopenia in hospitalized patients with lymphoma

In this single-center hospital-based cohort study of hospitalized patients with lymphoma, CT-defined sarcopenia was identified in 24.26% of the cohort. This proportion is clinically meaningful because the present study defined sarcopenia according to the coexistence of reduced CT-derived skeletal muscle mass and low handgrip strength. This combined definition requires impairment in both muscle quantity and muscle strength and therefore reflects a more clinically specific sarcopenia phenotype (12). The finding that nearly one quarter of hospitalized patients with lymphoma met this definition indicates a substantial burden of sarcopenia in this population.

Direct comparisons with previous studies should be made cautiously because lymphoma-specific evidence remains limited and heterogeneous. Available studies have mainly focused on diffuse large B-cell lymphoma or selected hematologic malignancies, and sarcopenia has commonly been assessed using radiologically measured muscle mass, skeletal muscle density, psoas muscle index, or other body composition parameters, with less consistent incorporation of muscle strength assessment (16–18). Differences in lymphoma subtype, treatment setting, imaging modality, diagnostic cut-off values, age distribution, and hospitalization status may all contribute to variation in reported prevalence. Accordingly, the present findings should be interpreted as evidence that clinically defined sarcopenia is common among hospitalized patients with lymphoma, instead of being reduced to a simple numerical comparison with estimates derived from other malignancies or different diagnostic criteria.

This observation has important clinical implications. Patients with lymphoma often undergo intensive systemic therapy, repeated hospitalization, and periods of reduced food intake, systemic inflammation, and functional decline, all of which may contribute to muscle loss and impaired muscle function. However, sarcopenia is not routinely assessed in many lymphoma care settings, and its clinical burden may have been insufficiently recognized. By integrating CT-derived skeletal muscle index with handgrip strength, the present study provides a clinically relevant assessment of sarcopenia and further links this phenotype to nutritional vulnerability and short-term adverse clinical outcomes. These findings support greater attention to muscle assessment in hospitalized patients with lymphoma, particularly in those with reduced intake, poor nutritional reserve, and impaired treatment tolerance.

### 4.2 Nutritional and clinical factors associated with CT-defined sarcopenia

The adjusted analyses in the present study suggest that nutritional compromise was more consistently associated with CT-defined sarcopenia than several other clinical variables. In the base clinical model, lower body mass index and reduced food intake during the previous week were significantly associated with sarcopenia. In the nutritional extension model, GNRI below 98 and reduced food intake during the previous week remained significantly associated with sarcopenia, and the overall model fit was better than that of the base clinical model. These findings indicate that recent dietary deterioration and poorer nutritional reserve may be particularly relevant to the identification of sarcopenia in hospitalized patients with lymphoma.

The association between lower body mass index and sarcopenia is biologically plausible and is broadly consistent with previous evidence linking malnutrition and sarcopenia (19). Lower body mass index often reflects reduced energy reserve and inadequate nutritional support for the maintenance of lean tissue. Reduced food intake during the previous week also showed a stable association with sarcopenia across models, which is clinically meaningful because recent deterioration in intake is relatively easy to identify in routine practice and may indicate ongoing muscle depletion (20–22). This interpretation is also supported by evidence showing that recent and current low food intake is common and clinically relevant among hospitalized patients across medical specialties (23) In addition, broader dietary quality has also been linked to sarcopenia risk, with prospective cohort evidence showing that greater adherence to the Mediterranean diet is associated with a lower risk of incident sarcopenia (40).

By contrast, ECOG performance status of 2 or higher, type 2 diabetes mellitus, and hypertension did not remain statistically significant after adjustment in the present models, although their effect estimates suggested possible clinical relevance. Poorer functional status may still be related to sarcopenia because reduced activity, functional decline, and nutritional vulnerability often coexist in patients with cancer (24, 25). Similarly, previous studies have suggested potential links between type 2 diabetes mellitus and sarcopenia through insulin resistance, oxidative stress, and chronic low-grade inflammation (26–28), while hypertension may also be associated with skeletal muscle decline through vascular dysfunction and impaired muscle perfusion (29, 30). In the present study, however, these variables did not retain statistical significance after adjustment, which may be related to the limited sample size and the modest number of sarcopenia events, as well as overlap between these clinical variables and the nutritional indicators included in the models.

Overall, the present findings suggest that when the analysis is restricted to a more parsimonious framework, nutritional variables contribute more consistently to the identification of CT-defined sarcopenia than several broader clinical factors. These findings support evaluating body composition together with nutritional vulnerability in hospitalized patients with lymphoma.

### 4.3 Auxiliary discriminatory performance of nutritional and screening indicators

An important extension of the present study is the comparison of several clinically accessible indicators for the identification of CT-defined sarcopenia. Among the evaluated indicators, GNRI yielded the numerically highest AUC, while PG-SGA showed a closely similar level of discriminatory performance. SARC-F and calf circumference had moderate discriminatory ability, whereas PNI performed less well in this cohort.

The relatively favorable performance of GNRI is clinically understandable. GNRI is derived from serum albumin and body weight and therefore reflects both nutritional reserve and systemic condition (9–11). In the present study, its optimal cut-off value was close to 98, which is also consistent with its performance in the multivariable model. These findings support the potential value of GNRI as a simple and practical indicator for identifying patients with a higher likelihood of CT-defined sarcopenia. This interpretation is also broadly consistent with previous evidence showing that GNRI is clinically relevant in lymphoma, including its association with prognosis in diffuse large B-cell lymphoma (31). PG-SGA also showed good discriminatory ability. As a multidimensional nutritional assessment tool, PG-SGA captures recent changes in intake, weight, symptoms, and functional impairment, which may explain its close relationship with sarcopenia in hospitalized patients with lymphoma.

By contrast, SARC-F and calf circumference showed only moderate performance. These indicators remain clinically useful because they are easy to obtain at the bedside and require minimal resources, although their AUCs were lower than those of GNRI and PG-SGA in the present cohort. PNI showed relatively weak discriminatory ability. This suggests that, although PNI reflects nutritional and immune status, it may be less sensitive than GNRI for identifying CT-defined sarcopenia in this clinical setting. Taken together, these findings suggest that GNRI and PG-SGA may provide useful adjunctive information for identifying patients who warrant further assessment of muscle mass and strength.

### 4.4 Relationships of GNRI and PNI with SMI and handgrip strength

Another notable finding is that GNRI was positively correlated with both skeletal muscle index and handgrip strength, whereas PNI was not significantly correlated with either measure. This result is important because it suggests that the value of GNRI is not limited to the binary classification of sarcopenia. Instead, GNRI also showed meaningful relationships with both muscle mass and muscle strength, which are the two core domains underlying the diagnosis of sarcopenia.

This pattern further supports the role of GNRI as a clinically informative nutritional indicator in patients with lymphoma. A lower GNRI may reflect reduced protein and energy reserve, poorer general condition, and a metabolic environment that is less favorable for the preservation of skeletal muscle (10, 11, 32, 33). The weaker performance of PNI in both the receiver operating characteristic analysis and the correlation analysis suggests that not all nutrition-related indices have the same usefulness for the evaluation of CT-defined sarcopenia. In practical terms, GNRI appears to provide more stable and clinically relevant information than PNI in the present cohort.

### 4.5 Coexistence of sarcopenia and malnutrition and short-term clinical burden

The coexistence of CT-defined sarcopenia and GLIM-defined malnutrition was another clinically meaningful finding of the present study. Although sarcopenia and malnutrition are conceptually distinct, they often share common drivers in hospitalized patients with lymphoma, including reduced food intake, systemic inflammation, treatment-related symptoms, physical inactivity, and disease burden (34, 35). In the present cohort, approximately two thirds of patients with sarcopenia also met the criteria for GLIM-defined malnutrition, and the joint analysis further showed that patients with both conditions had particularly high rates of infection or febrile neutropenia and prolonged hospitalization.

In patients with lymphoma, treatment tolerance depends not only on tumor characteristics and treatment regimen, but also on the capacity of the host to withstand metabolic stress, infection, and repeated hospitalization. The observation that treatment tolerance events were frequent in both sarcopenia groups is also noteworthy. It suggests that CT-defined sarcopenia itself may capture aspects of vulnerability that are not fully explained by GLIM-defined malnutrition or GNRI. This interpretation is consistent with previous evidence indicating that sarcopenia in patients with cancer is associated with adverse clinical outcomes and may reflect a broader decline in nutritional and functional reserve (5, 6, 36). Therefore, CT-based body composition assessment may provide complementary information beyond conventional nutritional evaluation.

The attenuation of infection-related associations after additional adjustment for nutritional status suggests that impaired nutritional reserve may partly explain these outcomes. In contrast, the association with treatment tolerance events remained significant, indicating that CT-defined sarcopenia may capture aspects of functional and treatment-related vulnerability beyond conventional nutritional assessment. Protein-energy insufficiency, low food intake, and systemic inflammation may all contribute to muscle loss and reduced host reserve, which provides a plausible biological background for the observed associations (20–23, 35, 37, 38). These results support a more integrated approach in hospitalized patients with lymphoma, in which CT-based muscle assessment, nutritional evaluation, and clinical risk monitoring are considered together rather than separately.

The present study has several strengths. CT-defined sarcopenia was present in nearly one quarter of the cohort and was associated with nutritional vulnerability, impaired functional reserve, and a higher short-term clinical burden. The study also compared several simple indicators that can be obtained in routine practice and extended the analysis to short-term clinical outcomes related to infection, treatment delivery, and hospitalization burden. Several limitations should also be acknowledged. First, this was a single-center hospital-based cohort study that included only hospitalized patients with lymphoma, which may limit the generalizability of the findings to outpatients, other institutions, or patients with different disease severity. Second, the sample size was relatively limited, and the number of sarcopenia events was modest, which may have affected the stability of multivariable estimates. Third, detailed treatment phase and line of therapy were not prospectively collected in a standardized manner and therefore could not be reliably incorporated into the adjusted analyses. Fourth, some laboratory and clinical variables were incomplete, and no external validation cohort was available. Finally, this study focused on short-term clinical outcomes; longer follow-up is needed to clarify the prognostic implications of CT-defined sarcopenia for survival, treatment response, and long-term functional recovery. Future multicenter studies with larger samples and longitudinal designs are needed to confirm these findings and to determine whether early nutritional and functional interventions can improve clinically meaningful outcomes in patients with lymphoma (36, 39).

## 5 Conclusion

In hospitalized patients with lymphoma, CT-defined sarcopenia was common and was associated with nutritional compromise and reduced functional reserve. Lower body mass index, reduced recent food intake, and GNRI below 98 were associated with a higher likelihood of sarcopenia, while GNRI and PG-SGA showed comparable and relatively favorable auxiliary discriminatory performance among the evaluated indicators.

CT-defined sarcopenia was also associated with a higher short-term clinical burden, including infection or febrile neutropenia, in-hospital infection, treatment tolerance events, treatment interruption, and longer cumulative hospitalization. The association with treatment tolerance events remained relatively stable after additional adjustment for nutritional status, suggesting that CT-defined sarcopenia may provide clinical information beyond routine nutritional assessment.

The frequent coexistence of sarcopenia and GLIM-defined malnutrition supports the clinical relevance of integrating CT-based body composition assessment with nutritional and functional evaluation in hospitalized patients with lymphoma. Future multicenter longitudinal studies are warranted to confirm these associations and to clarify whether early identification and intervention can improve treatment delivery and clinical outcomes.

## Data Availability

The datasets presented in this article are not readily available because they contain patient-level clinical information derived from medical records, nutritional assessments, computed tomography-based skeletal muscle analysis, laboratory tests, and short-term inpatient outcome records. Public sharing is restricted because the data contain potentially identifiable and sensitive health information and are subject to patient privacy protections and institutional ethical requirements. Access to de-identified data may be considered upon reasonable request to the corresponding author, subject to appropriate institutional approval and in accordance with applicable ethical and privacy regulations. Requests to access the datasets should be directed to Xingyue Zhai, xingyue@dmu.edu.cn.
