# Supplementary material for: Computed tomography-defined sarcopenia is associated with nutritional vulnerability and short-term clinical burden in hospitalized patients with lymphoma
Source: Front Nutr. 2026 Aug 17;13:1920154. doi: 10.3389/fnut.2026.1920154 (PMC13526627; doi:10.3389/fnut.2026.1920154)
Supplement: Supplementary file 1 [file Table_1.DOCX]

**Supplementary Table S1. Sensitivity analyses of the associations between CT-defined sarcopenia and short-term adverse clinical outcomes after additional adjustment for nutritional status**

| Outcome | Model | Additional adjustment | Adjusted OR | 95% CI | P value | N | Events |
| --- | --- | --- | --- | --- | --- | --- | --- |
| Infection or febrile neutropenia | Model 3 | Additionally adjusted for GNRI | 2 | 0.81–4.95 | P = 0.133 | 135 | 60 |
| Infection or febrile neutropenia | Model 4 | Additionally adjusted for GLIM-defined malnutrition | 1.92 | 0.75–4.90 | P = 0.172 | 136 | 60 |
| In-hospital infection | Model 3 | Additionally adjusted for GNRI | 1.9 | 0.78–4.63 | P = 0.158 | 135 | 46 |
| In-hospital infection | Model 4 | Additionally adjusted for GLIM-defined malnutrition | 1.75 | 0.69–4.48 | P = 0.241 | 136 | 46 |
| Treatment tolerance event | Model 3 | Additionally adjusted for GNRI | 3.38 | 1.41–8.13 | P = 0.006 | 135 | 41 |
| Treatment tolerance event | Model 4 | Additionally adjusted for GLIM-defined malnutrition | 2.54 | 1.02–6.34 | P = 0.046 | 136 | 42 |
| Treatment interruption | Model 3 | Additionally adjusted for GNRI | 2.54 | 0.97–6.68 | P = 0.059 | 135 | 27 |
| Treatment interruption | Model 4 | Additionally adjusted for GLIM-defined malnutrition | 1.71 | 0.61–4.83 | P = 0.311 | 136 | 28 |
| Prolonged hospitalization >60 days | Model 3 | Additionally adjusted for GNRI | 2.48 | 1.02–6.03 | P = 0.046 | 135 | 46 |
| Prolonged hospitalization >60 days | Model 4 | Additionally adjusted for GLIM-defined malnutrition | 1.43 | 0.56–3.63 | P = 0.457 | 136 | 47 |
