# Supplementary material for: Computed tomography-defined sarcopenia is associated with nutritional vulnerability and short-term clinical burden in hospitalized patients with lymphoma
Source: Front Nutr. 2026 Aug 17;13:1920154. doi: 10.3389/fnut.2026.1920154 (PMC13526627; doi:10.3389/fnut.2026.1920154)
Supplement: Supplementary file 2 [file Table_2.docx]

Supplementary Table 2. STROBE Statement—Checklist for Cohort Studies

Frontiers in Nutrition

| **Manuscript title** | Computed tomography-defined sarcopenia is associated with nutritional vulnerability and short-term clinical burden in hospitalized patients with lymphoma |
| --- | --- |
| **Study design** | Single-center cohort study |
| **Article type** | Original Research |
| **Journal/section** | Frontiers in Nutrition / Clinical Nutrition |
| **Prepared for** | Publication as Supplementary Table 2 |

Note. This checklist was completed according to the STROBE recommendations for cohort studies. Locations are reported using manuscript section headings and table or supplementary material identifiers. Page and line numbers have been omitted because they may change during production. Where an item is not applicable, a brief explanation is provided.

| **Section** | **Item** | **STROBE recommendation** | **Status** | **Location / notes** |
| --- | --- | --- | --- | --- |
| Title and abstract | 1a | Indicate the study design with a commonly used term in the title or abstract. | Yes | Title and Abstract; the Abstract identifies the study as a single-center cohort study. |
|  | 1b | Provide an informative and balanced summary of what was done and what was found. | Yes | Abstract; includes the study population, sarcopenia definition, main nutritional findings, ROC results, and short-term outcome associations. |
| Introduction | 2 | Explain the scientific background and rationale for the investigation being reported. | Yes | Introduction; explains lymphoma burden, nutritional vulnerability, sarcopenia, and the evidence gap. |
|  | 3 | State specific objectives, including any prespecified hypotheses. | Yes | Introduction, final paragraph; states the objectives regarding prevalence, associated factors, discriminatory indicators, and short-term outcomes. |
| Methods | 4 | Present key elements of study design early in the paper. | Yes | Materials and methods—Participants; presents the single-center cohort design, baseline assessment, and subsequent ascertainment of short-term outcomes. |
|  | 5 | Describe the setting, locations, and relevant dates, including periods of recruitment, exposure, follow-up, and data collection. | Yes | Materials and methods—Participants; describes the Lymphoma Ward of the Second Hospital of Dalian Medical University, recruitment dates, and the predefined observation period. |
|  | 6a | Give the eligibility criteria, and the sources and methods of selection of participants. Describe methods of follow-up. | Yes | Materials and methods—Participants and Assessment of Short-Term Clinical Outcomes; eligibility criteria, participant selection, and outcome ascertainment are described. |
|  | 6b | For matched studies, give matching criteria and number of exposed and unexposed. | Not applicable | No matching design was used. |
|  | 7 | Clearly define all outcomes, exposures, predictors, potential confounders, and effect modifiers. Give diagnostic criteria, if applicable. | Yes | Materials and methods—Assessment of Sarcopenia, Measurement of Covariates, Laboratory Tests, and Assessment of Short-Term Clinical Outcomes; diagnostic criteria, predictors, covariates, and outcomes are defined. |
|  | 8 | For each variable of interest, give sources of data and details of methods of assessment or measurement. Describe comparability of assessment methods if more than one group. | Yes | Materials and methods—Assessment of Sarcopenia, Measurement of Covariates, Laboratory Tests, and Assessment of Short-Term Clinical Outcomes; data sources and measurement methods are described. |
|  | 9 | Describe any efforts to address potential sources of bias. | Partly reported | Materials and methods—Participants, Assessment of Sarcopenia, Assessment of Short-Term Clinical Outcomes, and Statistical Analysis; residual confounding is acknowledged in the Discussion limitations. |
|  | 10 | Explain how the study size was arrived at. | Yes | Materials and methods—Sample Size Estimation; the prevalence-based calculation and final target sample size are reported. |
|  | 11 | Explain how quantitative variables were handled in the analyses. If applicable, describe which groupings were chosen and why. | Yes | Materials and methods—Statistical Analysis; handling of continuous and categorical variables and prespecified category boundaries is described. |
|  | 12a | Describe all statistical methods, including those used to control for confounding. | Yes | Materials and methods—Statistical Analysis; group comparisons, logistic regression, ROC analysis, Spearman correlation, covariate adjustment, and sensitivity models are reported. |
|  | 12b | Describe any methods used to examine subgroups and interactions. | Yes | Materials and methods—Statistical Analysis; exploratory joint groups defined by CT-based sarcopenia and GLIM-defined malnutrition are described. |
|  | 12c | Explain how missing data were addressed. | Partly reported | Materials and methods—Statistical Analysis; analyses were performed on available cases. A detailed missingness table was not included. |
|  | 12d | If applicable, explain how loss to follow-up was addressed. | Partly applicable | Materials and methods—Participants and Assessment of Short-Term Clinical Outcomes; outcomes were ascertained from inpatient records and follow-up information. No formal loss-to-follow-up analysis was reported. |
|  | 12e | Describe any sensitivity analyses. | Yes | Materials and methods—Statistical Analysis; Results—CT-defined Sarcopenia and Short-Term Adverse Clinical Outcomes; Supplementary Table S1. |
| Results | 13a | Report numbers of individuals at each stage of study, for example potentially eligible, examined for eligibility, confirmed eligible, included in the study, completing follow-up, and analyzed. | Partly reported | Results—Study Population Characteristics; the final cohort of 136 analyzed participants is reported. A detailed participant flow diagram was not provided. |
|  | 13b | Give reasons for non-participation at each stage. | Partly reported | Materials and methods—Participants; exclusion criteria are described, although numbers excluded at each stage are not separately reported. |
|  | 13c | Consider use of a flow diagram. | Not included | No flow diagram was included. This may be added if requested by the journal. |
|  | 14a | Give characteristics of study participants and information on exposures and potential confounders. | Yes | Results—Study Population Characteristics and Table 1; demographic, clinical, nutritional, and comorbidity characteristics are reported. |
|  | 14b | Indicate number of participants with missing data for each variable of interest. | Partly reported | Materials and methods—Statistical Analysis; analyses were performed on available cases. Detailed missing-data counts are not presented for each variable. |
|  | 14c | Summarize follow-up time, for example average and total amount. | Partly applicable | Materials and methods—Assessment of Short-Term Clinical Outcomes; Results—Short-Term Clinical Outcomes According to CT-defined Sarcopenia; Table 5. |
|  | 15 | Report numbers of outcome events or summary measures over time. | Yes | Results—Short-Term Clinical Outcomes According to CT-defined Sarcopenia, CT-defined Sarcopenia and Short-Term Adverse Clinical Outcomes, and Joint Clinical Burden of CT-defined Sarcopenia and GLIM-defined Malnutrition; Tables 5–7. |
|  | 16a | Give unadjusted and, if applicable, confounder-adjusted estimates and their precision. Make clear which confounders were adjusted for and why they were included. | Yes | Materials and methods—Statistical Analysis; Results—Short-Term Clinical Outcomes According to CT-defined Sarcopenia and CT-defined Sarcopenia and Short-Term Adverse Clinical Outcomes; Tables 5 and 6. |
|  | 16b | Report category boundaries when continuous variables were categorized. | Yes | Materials and methods—Assessment of Sarcopenia, Measurement of Covariates, Assessment of Short-Term Clinical Outcomes, and Statistical Analysis; category boundaries are reported. |
|  | 16c | If relevant, consider translating estimates of relative risk into absolute risk for a meaningful time period. | Partly applicable | Tables 5 and 7 provide absolute event rates; adjusted associations are reported as odds ratios. |
|  | 17 | Report other analyses done, for example analyses of subgroups and interactions, and sensitivity analyses. | Yes | Materials and methods—Statistical Analysis; Results—CT-defined Sarcopenia and Short-Term Adverse Clinical Outcomes and Joint Clinical Burden of CT-defined Sarcopenia and GLIM-defined Malnutrition; Supplementary Table S1. |
| Discussion | 18 | Summarize key results with reference to study objectives. | Yes | Discussion, opening paragraph, and Conclusions. |
|  | 19 | Discuss limitations of the study, taking into account sources of potential bias or imprecision. | Yes | Discussion, limitations paragraph; the single-center design, hospitalized population, limited sample size, modest number of sarcopenia events, incomplete variables, treatment-phase limitations, and lack of external validation are acknowledged. |
|  | 20 | Give a cautious overall interpretation of results considering objectives, limitations, multiplicity of analyses, similar studies, and other relevant evidence. | Yes | Discussion, Sections 4.1–4.5, and Conclusions; findings are interpreted as associations rather than causal effects. |
|  | 21 | Discuss the generalisability of the study results. | Partly reported | Discussion, limitations paragraph; generalizability to outpatients, other institutions, and patients with different disease severity is addressed cautiously. |
| Other information | 22 | Give the source of funding and the role of the funders for the present study. | Yes | Funding statement; the funder, grant number, and absence of a funder role in study design, data collection, analysis, interpretation, manuscript preparation, and submission are reported. |

**Abbreviations:** CT, computed tomography; GLIM, Global Leadership Initiative on Malnutrition; GNRI, Geriatric Nutritional Risk Index; ROC, receiver operating characteristic; SMI, skeletal muscle index; STROBE, Strengthening the Reporting of Observational Studies in Epidemiology.
